# The Optimal Dosing Regimen of Super Bioavailable Itraconazole in Obesity: An Experimental Rat Model Study

**DOI:** 10.7759/cureus.37462

**Published:** 2023-04-11

**Authors:** Gaurav K Jain, Kruttika R Chitnis, Payal Singhal, Namrata Mahadkar, Dhiraj Dhoot, Hanmant Barkate

**Affiliations:** 1 Centre for Advanced Formulation Technology, Delhi Institute of Pharmaceutical Sciences and Research, New Delhi, IND; 2 Global Medial Affairs, Glenmark Pharmaceuticals Limited, Mumbai, IND

**Keywords:** serum concentration, fatty tissue concentration, skin concentration, obesity, superficial fungal infections, dermatophytosis, super-bioavailable itraconazole

## Abstract

Background

Obesity may alter tissue distribution and clearance of several drugs, especially lipophilic ones. Itraconazole, a lipophilic drug, has been recently introduced in a super-bioavailable formulation (SB-ITZ) for the treatment of dermatophytosis. Evidence regarding optimal dosing of SB-ITZ in obesity is lacking. A current experimental study was planned to analyze tissue concentrations of SB-ITZ at different doses in obese and non-obese rats.

Materials and methods

Thirty-six Wistar albino rats of either sex were divided into obese and non-obese rats equally. Further, rats in both categories were divided into three dosing groups. Group 1 received SB-ITZ 13 mg once daily in the morning, group 2 received SB-ITZ 13 mg in the morning and 6.5 mg in the evening, while Group 3 rats received SB-ITZ 13 mg twice daily, orally. Concentrations of SB-ITZ in the skin, serum, and fatty tissue were assessed in each group on days 7, 14, 21, and 28. Comparison of SB-ITZ concentrations in various tissues in obese and non-obese rats and inter-group comparison of tissue concentrations across the three dosing regimens was done at day 28 and expressed as Mean ± SD.36 Wistar rats were divided into obese and non-obese rats equally.

Results

At day 28, skin concentrations of SB-ITZ were 5.36±1.1, 8.9±1.7 and 10.13±1.7 µg/g in Groups 1, 2, and 3, respectively, in non-obese rats, which was statistically significant (p<0.05) than skin concentration of obese rats (2.72±0.6, 4.2±0.7 and 4.66±0.5 µg/g) for the corresponding dosing groups respectively. Skin concentration of SB-ITZ was statistically significant for Groups 2 and 3 as compared to Group 1. Still, no statistically significant difference was noted between Groups 2 and 3 in non-obese and obese rats. Fatty tissue concentration of SB-ITZ was comparable in all 3 dosing regimens in non-obese and obese rats. But on the intergroup comparison, a statistically significant difference was observed for Groups 2 and 3 against Group 1 (p<0.05). Increasing the dose of SB-ITZ increased serum concentration. In non-obese rats, a statistically significant difference was noted between Group 2 (74.33±6.6 ng/ml) and Group 1 (52.5±9.9 ng/ml); p<0.01 and also in Group 3 (81.33±6.8 ng/ml) against Group 1; p<0.01. Group 3 achieved significantly higher concentration than the other two groups in obese rats (Group 3; 72±5.3, Group 2; 60.5±4.3, and Group 1; 45±7 ng/ml; p<0.01).

Conclusion

Overall, skin, fatty tissue, and serum concentrations of SB-ITZ were higher in non-obese rats compared to obese rats in all three dosing groups. Moreover, skin and fatty tissue concentrations were proportionately higher than serum in all the groups in non-obese and obese rats. Though the skin concentration of non-obese rats was significantly higher than obese rats, skin concentration in obese rats was within the minimum inhibitory concentration (MIC) range, demonstrating the efficacy of all dosing regimens.

## Introduction

Dermatophytosis has reached epidemic proportions in India, with a staggering prevalence rate of 37-74%, with an increase in recalcitrant cases [1]. Many risk factors have been identified for this change, and obesity has been regarded as one of the important risk factors [1-5]. Owing to these changes, the current management of dermatophytosis has evolved as combination therapy, including topical and systemic antifungals and itraconazole, the most commonly prescribed systemic antifungal drug [6]. However, due to pharmacokinetic obstacles like food dependency, a requirement of an acidic environment for optimal absorption, etc., presented by conventional formulation of itraconazole, a newer itraconazole formulation, super-bioavailable itraconazole (SB-ITZ) was recently approved by Central Drugs Standard Control Organization (CDSCO) in India [7,8].

In obesity, the pharmacokinetics of lipophilic drugs is altered in tissue distribution and clearance of drugs, resulting in varying practices of initiation and maintenance doses of several drugs [9,10]. As a result, higher dosages of itraconazole have been utilized in treating severe mycosis and recalcitrant dermatophytosis worldwide, especially in obese patients [1,11,12]. In one of the studies, it was mentioned that the dose of itraconazole should be doubled in obese patients as compared to non-obese patients with dermatophytosis [13].

Hence, it is imperative to identify the concentration of SB-ITZ in different tissues like fats and skin at different doses. However, evidence regarding the optimal dosing of SB-ITZ in obese dermatophytosis patients is lacking. Thus this experimental study was planned to compare different tissue concentrations of SB-ITZ at different doses and time intervals in obese and non-obese rats.

## Materials and methods

Ethical considerations

The study protocol was approved by the Institutional Animal Ethics Committee of Delhi Pharmaceutical Sciences and Research University [DPSRU (IAEC/2022/II-R05)], registered under CPCSEA (215/Go/ReBi/2000/CPCSEA). The study was performed at Delhi Pharmaceutical Sciences and Research University per the guidelines of the Committee for Control and Supervision of Experiments on Animals (CPCSEA), India.

Animals

Thirty-six Wistar Albino rats (Sp. Rattus norvegicus) of 6 weeks’ age of either sex with baseline weight range 135±8 gm were housed in a climate-controlled room (22 ± 2°C and 60% relative humidity) with a 12 h light/dark cycle. Initially, animals were randomly divided into two equal groups. Rats in both groups had ad libitum access to water and food, but Group 1 rats (n=18) received the standard diet (non-obese group) while Group 2 rats (n=18) received the high-fat diet (obese group) for 2 months or until weight gain is more than 50%. Later, the non-obese and obese rats were randomly divided into 3 equal groups, with 6 non-obese and 6 obese rats in each group (n=6) receiving different doses of itraconazole. 

Chemicals

Super-bioavailable itraconazole (SB-ITZ) was provided by Glenmark Pharmaceuticals Ltd (Mumbai, India). All other reagents and solvents used were of analytical grade and were purchased from Qualigens Fine Chemicals (Mumbai, India).

Dose and administration

Rat doses were calculated from human equivalent doses [14]. They were dosed orally by dispersing the weighed amount of SB-ITZ into 1.0 ml of water and feeding the animals through oral feeding sonde. Group 1 rats received SB-ITZ 13 mg once daily in the morning, Group 2 received SB-ITZ 13 mg in the morning and 6.5 mg in the evening, while Group 3 received SB-ITZ 13 mg twice daily after food. All the groups received treatment for 4 weeks.

Tissue preparation

To determine ITZ concentration in serum, blood was collected from the lateral tail vein of a rat under local anesthesia using a 23-gauge needle. The plasma was separated from blood by the standard procedure. To determine SB-ITZ concentration in skin and fatty tissue, the animals were anesthetized, and 6 cm paravertebral incisions were made through the full thickness of the skin using a skin biopsy punch. Using a scalpel, the skin was carefully separated from fatty tissue from the excised tissue. The skin and fatty tissues were separately homogenized in methanol, and samples were filtered and stored at -20°C until the analysis was performed [15].

Concentration analysis method

As reported previously, the concentration of SB-ITZ in plasma and tissue samples was determined by High-performance liquid chromatography (HPLC) [6]. Briefly, HPLC (LC-10 AT VP, Shimadzu Corp., Kyoto, Japan) coupled with UV-Vis Detector (SPD-10A VP, Shimadzu Corp., Kyoto, Japan) was used to determine ITZ. Mobile Phase consisted of acetonitrile and 0.05% diethylamine in a ratio of 60:40 (v/v). Chromatographic separation was performed using a C18 column (Phenomenex C-18, 4.6 × 250 mm, 5 μm) at a 1 mL/min flow rate with a detection wavelength of 258 nm. An aliquot of 1.0 mL from each plasma or tissue sample was mixed with 2.0 mL of acetonitrile, followed by vortex mixing for 5 min. The mixture was then subjected to centrifugation at 10,000 rpm for 10 min. The organic layer was separated and dried under a gentle stream of nitrogen and reconstituted with a 200 µL mobile phase. A 100 µL of the reconstituted sample was injected into the HPLC column. The limit of quantification of the developed HPLC method was 2.0 ng/mL for ITZ. The HPLC graphs were obtained, and the area was converted into concentration accordingly. 

Parameters and timing of assessment

Skin, fatty tissue, and serum concentrations of SB-ITZ were determined on days 7, 14, 21, and 28.

Statistical analysis

The data were expressed as mean ± standard deviation. Tissue concentrations in non-obese and obese rats were analyzed using the student’s T-test. Inter-group comparison of tissue concentrations amongst 3 dosing groups was made using one-way ANOVA. P-value <0.05 was considered statistically significant.

**Figure 1:**
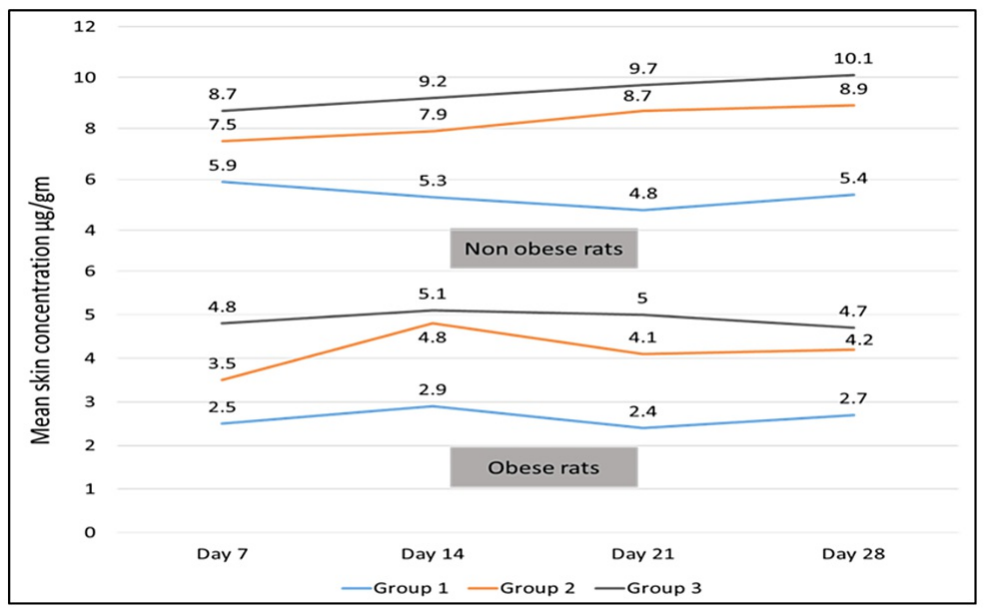
Skin concentration of super-bioavailable itraconazole (µg/gm) in non-obese and obese rats for 3 dosing regimens at different time intervals

**Figure 2:**
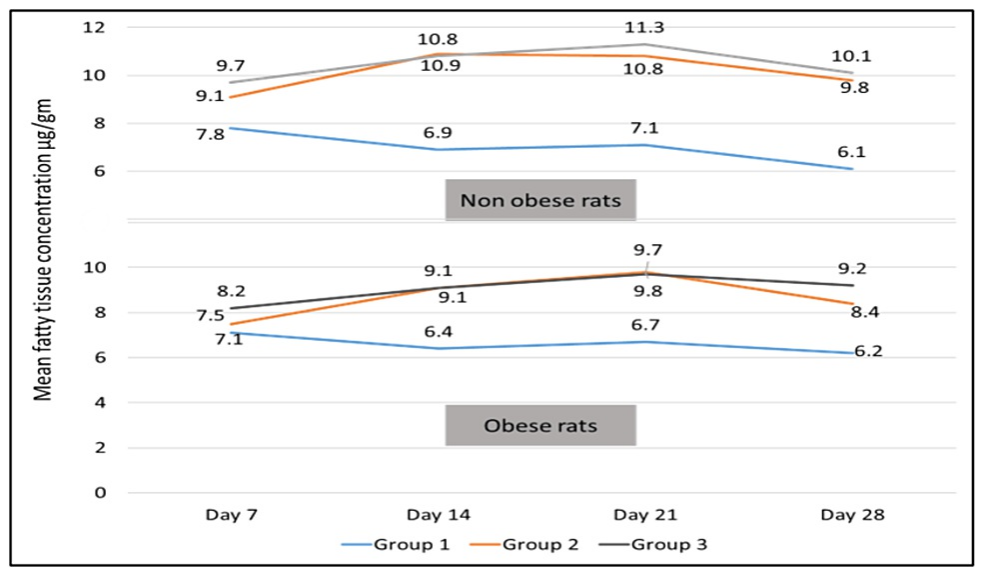
Fatty tissue concentration of super-bioavailable itraconazole (µg/gm) in non-obese and obese rats for 3 dosing regimens at different time intervals

**Figure 3:**
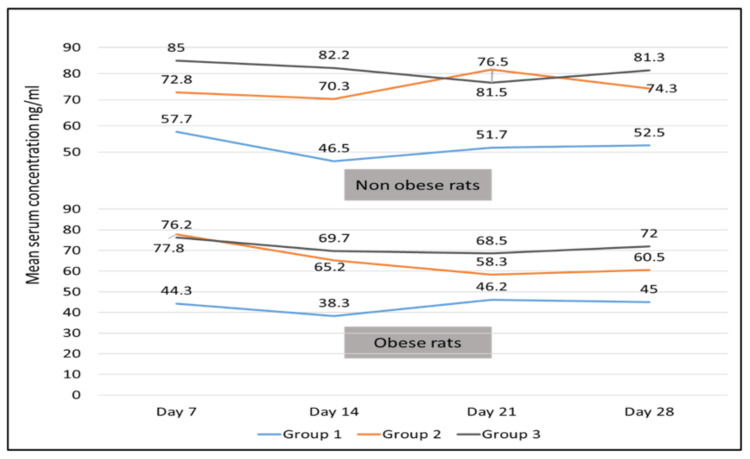
Serum concentration of super-bioavailable itraconazole (ng/ml) in non-obese and obese rats for 3 dosing regimens at different time intervals

Mean skin, fatty tissue, and serum concentrations for all three groups are depicted in Figures 1, 2, and 3. But owing to the chronicity of the disease and duration of therapy, results of day 28 only were considered for further analysis.

## Results

Skin concentration

Skin concentration of SB-ITZ was highest in Group 3 in non-obese and obese rats at day 28. The difference between skin concentrations of non-obese rats and obese rats was highly statistically significant in all 3 groups (p<0.05). Moreover, in obese rats, the inter-group analysis revealed a statistically significant difference in Group 1 versus 2 (p = 0.003) and Group 1 versus 3 (p = 0.0003). However, a statistically significant difference was not found in group 2 versus 3 analyses [(p = 0.97) (Table 1 and Fig 1)].

**Table 1:** Concentration of super bioavailable itraconazole (µg/gm) in skin of non-obese and obese rats in 3 dosing regimens at day 28 *highly significant; #significant

Group	Inter-group comparison	Non-obese rats (n=6); Mean±SD (µg/g)	Obese rats (n=6); Mean±SD (µg/g)	p-value (non-obese v/s obese)
1	-	5.36 ± 1.1	2.72 ± 0.6	<0.05*
2	-	8.9 ± 1.7	4.2 ± 0.7	<0.05*
3	-	10.13 ± 1.7	4.66 ± 0.5	<0.05*
p-value	1 v/s 2	<0.05#	<0.05#	-
	2 v/s 3	0.378	0.975	-
	1 v/s 3	<0.05*	<0.05*	-

Fatty tissue concentration

Amongst the 3 dosing regimens, the concentration of SB-ITZ in fatty tissue was highest in Group 3 in non-obese and obese rats at day 28. However, fatty tissue concentration of SB-ITZ was comparable in non-obese and obese rats in all 3 groups with no statistically significant difference, as shown in Table 2. Inter-group analysis revealed a statistically significant difference in Group 1 versus 2 (p = 0.02) and Group 1 versus 3 (p = 0.002) in obese rats. However, such a statistically significant difference was not found in Group 2 versus 3 (p = 0.52) analyses (Table 2 and Fig 2).

**Table 2:** Fatty tissue concentration of super bioavailable itraconazole (µg/gm) in non-obese and obese rats in 3 dosing regimens at day 28 *highly significant, #significant

Group	Inter-group comparison	Non-obese rats (n=6); Mean±SD (µg/g)	Obese rats (n=6); Mean±SD (µg/g)	p-value
1	-	6.06 ± 1.3	6.23 ± 1.0	0.815
2	-	9.8 ± 1.1	8.4 ± 1.6	0.121
3	-	10.11 ± 1.4	9.23 ± 1.0	0.243
p-value	1 v/s 2	<0.05*	<0.05#	-
	2 v/s 3	0.918	0.525	-
	1 v/s 3	<0.05*	<0.05*	-

Serum concentration

A statistically significant difference was noted between non-obese and obese rats for Groups 2 and 3 (p<0.05), however; no statistically significant difference was found in Group 1 (p = 0.16). Inter-group analysis revealed statistically significant differences for Group 3 versus Group 2, Group 3 versus Group 1 (p<0.05), and Group 1 versus 2 (p<0.001) in obese rats (Table 3 and Fig 3).

**Table 3:** Serum concentration of super bioavailable itraconazole (ng/ml) in non-obese and obese rats in 3 dosing regimens at day 28 *highly significant; #significant

Group	Inter-group comparison	Non-obese rats (n=6); Mean±SD (ng/ml)	Obese rats (n=6); Mean±SD (ng/ml)	p-value
1		52.50 ± 9.9	45.0 ± 7.0	0.162
2		74.33 ± 6.6	60.5 ± 4.3	<0.05*
3		81.33 ± 6.8	72.0 ± 5.3	0.05#
p-value	1 v/s 2	<0.05*	<0.05*	-
	2 v/s 3	0.308	<0.05*	-
	1 v/s 3	<0.05*	<0.05*	-

## Discussion

The duration of antifungal agent exposure inside the skin, nail, and nail bed is crucial in determining the efficacy of dermatophytosis therapy [16]. Itraconazole has a slow clearance from the skin and nails due to its high affinity to keratin. Therefore, it binds strongly in the stratum corneum, which is responsible for its high efficacy in dermatomycosis treatment [16]. Sobue et al. noted that the extent of itraconazole binding to skin keratin was 94-97%, which was several times higher compared to fluconazole and griseofulvin [16]. Owing to its lipophilicity and high volume of distribution, itraconazole is distributed into the skin in higher quantities than its plasma concentration [6].

However, the extra body weight in obese patients is likely due to the excess adipose tissue, and lipophilic molecules may preferentially accumulate to a higher extent in this tissue [9]. In the present study, skin concentration of super bioavailable itraconazole was significantly higher in non-obese rats than in obese rats across all three groups. This might be attributed to reduced local blood flow to adipose tissue in obese rats, thus affecting drug dissemination into the skin [7]. Since fat acts as a reservoir for lipophilic drugs like itraconazole, dissemination of the drug from fat to skin might be altered in obesity [17]. Another possible explanation for such finding might be extensive layers of subcutaneous fat in obese rats that need to be traversed to reach epidermal layers as compared to non-obese rats, wherein subcutaneous fat is less. This is relevant in humans as well since numerous studies have demonstrated a manifold increase in subcutaneous fat in obese subjects as compared to non-obese ones [18]. These findings might serve as a preliminary indicator for a higher dose requirement of SB-ITZ in obese dermatophytosis patients. There should be no second thought that these findings must be confirmed in human studies first. A similar conclusion was made by Anaissie et al. in their in vitro study on fluconazole, wherein it was recommended to use fluconazole in higher doses in obese patients [19].

The minimum inhibitory concentration (MIC) at the target site is one of the significant determinants of the efficacy of antifungal drugs. Shaw et al. [20] noted that the effective MIC of itraconazole against Trichophyton mentagrophytes was in the range of 7.8-1000 ng/ml. A similar range of MIC was reported in an in-vitro study by Van Cutsem et al. [21]. The skin concentration of SB-ITZ in all three dosing groups was well within this effective range in both non-obese and obese rats, thus giving a presumptive indicator of good efficacy.

The serum concentration of SB-ITZ in the present study was less in obese and non-obese than in skin and fatty tissue across all three groups. Itraconazole, a lipophilic molecule, is readily distributed to tissues rich in lipid content, and its retention in such tissues is good owing to tight tissue protein binding [22]. Thus tissue concentration of itraconazole is manifold higher compared to its plasma concentration. Similar findings were seen in studies by Korting et al. [2] and Heykant J. et al. [22], demonstrating ratios of tissue to plasma concentrations of itraconazole which ranged from 10:1 (liver) to >25:1 (fatty tissue), indicating good bioavailability in tissue to combat infection at the affected site.

In the present study, the concentration of SB-ITZ in fatty tissue was highest in group 3 in non-obese and obese rats; however, there was no statistical difference in non-obese versus obese rats across all 3 groups. In the current study, the mean skin concentration of SB-ITZ was comparable to fatty tissue concentration across all three dosing regimens for non-obese rats. In contrast, in obese rats, this difference was higher. Similar findings were reported by Sobue et al., wherein concertation of itraconazole in the subcutaneous fatty tissue of guinea pigs was compared to that of stratum corneum [16]. Itraconazole concentration in subcutaneous fatty tissue was 42 mcg/gm compared to 31.6 mcg/gm in stratum corneum [16]. This finding corroborates the earlier assumption that obese rats have thick subcutaneous fatty layers that act as drug reservoirs. The drug is released slowly to diffuse into epidermal layers, where it binds tightly to the stratum corneum after that. 

On analyzing the concentration of SB-ITZ in various tissues for the three dosing regimens, it was found that the difference in concentrations was highly statistically significant in Group 1 (SB-ITZ 13 mg OD) versus Group 2 (SB-ITZ 13 mg + 6.5 mg) and Group 1 versus 3 (SB-ITZ 13 mg BD) in non-obese as well as obese rats. This suggests that twice-daily doses of SB-ITZ 13mg and 13+6.5 mg will achieve significantly higher tissue concentrations than the once-daily dose. However, substantiation of the exact clinical benefit of such finding warrants human clinical trials.

The present study is the first attempt to generate documentary evidence for optimal dosing of SB-ITZ in obesity. Although this is a pre-clinical study, it might help to set a benchmark for dosing regimens in human studies, inclusive of evaluating skin concentration of SB-ITZ vis-à-vis MIC values against Trichophyton mentagrophytes and Trichophyton rubrum as the principal causative organism of dermatophytosis and the most sensitive to itraconazole amongst systemic antifungals in the current scenario.

## Conclusions

The present study is the first evidence of different super bioavailable itraconazole tissue concentrations in different dosing regimens in obese rats. Overall, fatty tissue and skin concentrations were proportionately higher as compared to serum in all three dosing groups in both obese as well as non-obese rats. However, skin concentration in obese and non-obese rats was within the MIC range. Thus, the optimal duration of therapy in obesity needs further consideration in improving outcomes in dermatophytosis patients. However, clinical studies are warranted to substantiate this evidence.
